# Diet and Lifestyle Changes During the COVID-19 Pandemic in Ibero-American Countries: Argentina, Brazil, Mexico, Peru, and Spain

**DOI:** 10.3389/fnut.2021.671004

**Published:** 2021-06-02

**Authors:** Oscar G. Enriquez-Martinez, Marcia C. T. Martins, Taisa S. S. Pereira, Sandaly O. S. Pacheco, Fabio J. Pacheco, Karen V. Lopez, Salomon Huancahuire-Vega, Daniela A. Silva, Ana I. Mora-Urda, Mery Rodriguez-Vásquez, M. Pilar Montero López, Maria C. B. Molina

**Affiliations:** ^1^Public Health Program, Health Sciences Center, Federal University of Espírito Santo, Vitória, Brazil; ^2^Center for Health Sciences Research, School of Medicine and Health Sciences, Universidad Adventista del Plata, Entre Ríos, Argentina; ^3^Institute for Food Science and Nutrition, Universidad Adventista del Plata, Entre Ríos, Argentina; ^4^Master in Science of Human Motricity, Adventist University of Chile, Chillán, Chile; ^5^Department of Health Sciences, Universidad de las Américas Puebla, San Andrés Cholula, Puebla, Mexico; ^6^Department of Basic Sciences, Faculty of Health Sciences, School of Human Medicine, Universidad Peruana Unión, Lima, Peru; ^7^Department of Integrated Health Education, Federal University of Espírito Santo, Vitória, Brazil; ^8^Biology Department, Universidad Autónoma de Madrid, Madrid, Spain; ^9^Health and Nutrition Program, Federal University of Ouro Preto, Ouro Preto, Brazil

**Keywords:** COVID-19, diet, lifestyle, confinement, pandemic, E-survey

## Abstract

This study aimed to evaluate changes in dietary and lifestyle habits during the period of confinement due to the first wave of the COVID-19 pandemic in Ibero-American countries. A cross-sectional investigation was conducted with 6,325 participants of both genders (68% women), over 18 years of age and from five countries: Brazil (*N* = 2,171), Argentina (*N* = 1,111), Peru (*N* = 1,174), Mexico (*N* = 686), and Spain (*N* = 1,183). Data were collected during the year 2020, between April 01 and June 30 in Spain and between July 13 and September 26, in the other countries studied using a self-administered online survey designed for the assessment of sociodemographic, employment, physical activity, health status, and dietary habits changes. Most participants (61.6%), mainly those from Spain, remained constant, without improving or worsening their pattern of food consumption. Among those who changed, a pattern of better eating choices prevailed (22.7%) in comparison with those who changed toward less healthy choices (15.7%). Argentina and Brazil showed the highest proportion of changes toward a healthier pattern of food consumption. Peruvians and Mexicans were less likely to make healthy changes in food consumption (OR: 0.51; 95% CI: 0.4–0.6 and OR: 0.69; 95% CI: 0.4–0.8, respectively), when compared to Argentinians. Most respondents did not change their pattern of meal consumption, but those who did reduced their consumption of main meals and increased intake of small meals and snacks. Although most participants affirmed to be doing physical activity at home, about one-half reported perception of weight gain. Individuals with alterations in sleep pattern (either by increasing or decreasing sleep time) were more likely to change their diets to a healthier pattern. In contrast, individuals with confirmed diagnosis of COVID-19 and those who reported feeling anxious were more likely to perform changes to a less healthy eating pattern (OR: 1.72; 95% CI: 1.2–2.3 and OR: 1.21; 95% CI: 1.1–1.4, respectively). In conclusion, although most participants remained constant in their eating habits, lifestyle changes and anxiety feelings were reported. Among those who changed patterns of food consumption, healthier choices prevailed, with differences between countries. However, there were alterations in the distribution of meals, with higher consumption of snacks and small meals. These results can be used to guide policies to prevent deleterious consequences that may affect the incidence of chronic diseases.

## Introduction

Despite global efforts, the rapid spread of the coronavirus disease 2019 (COVID-19) has strongly affected Ibero-American countries. Since the declaration of a world health emergency by the World Health Organization (1) in March 2020, followed by the declaration of the pandemic (2), each government has managed the situation using different strategies. The most common measures adopted varied from radical quarantine or total confinement; selective quarantine, meaning flexible confinement for part of the population and total confinement for vulnerable people (elderly and/or individuals with underlying diseases); to the maintenance of normality, aiming to obtain the so-called “herd immunity.”

While social physical distance practices are important to prevent the collapse of the health system, they also affect interpersonal relationships and many aspects of everyday life, including dietary and lifestyle habits of individuals, families, and populations. The pandemic now is far from being controlled and according to experts from the WHO and other institutions, we will not avoid future ones. As a result of this scenario, it is expected that physical and psychological consequences will last for a long time after the COVID-19 pandemics crisis. It can be expected that slowly evolving diet and lifestyle changes will affect the incidence of chronic diseases. Hence, it is difficult to estimate the extent of the consequences of lifestyle changes on the health system (3).

The impact of isolation, confinement, and social distancing in large populations has led to numerous statistical, educational, psychological, sociological, and historical studies, among others. Some have investigated how confinement affected people in their homes, the change in their routines and the consequent adaptations regarding eating habits, physical activity, use of screens, sleep patterns, among others (4–15). Many studies evaluated dietary changes (11, 12, 16). Some have focused on Chinese children and adolescents (7), Croatian adolescents and medical students (15), adults from Israel (17). Brazilian adults (11), single European countries (10, 18, 19) and children or adults with comorbidities (5, 6, 8). One study compared dietary changes among adolescents from different Ibero-American countries during the pandemic (9). Thus far, there are no comparative studies among adults from Ibero-American countries.

Understanding the effects of the pandemic on lifestyle habits can guide behavioral and psychological measures, directed at individuals and communities, improving resilience, preventing diseases, and increasing the effectiveness of health approaches to mitigate the effects during this contingency and in others that could potentially occur. Therefore, we aimed to assess changes in dietary and lifestyle habits due to COVID-19-induced confinement in different Ibero-American countries.

## Materials and Methods

### Study Design and Population

This study was a cross-sectional, online Ibero-American survey of adult men and women aged ≥ 18 years, from four Latin American countries (Argentina, Brazil, Mexico, and Peru) and one European country (Spain). Data were collected in the year 2020, between April 01 and June 30 in Spain and between July 13 and September 26, in Latin American countries, during the period of confinement due to the COVID-19 pandemic. This was a period of self-isolation, remote work, and restrictions of access to indoor and outdoor places. Participants were invited mainly through the research team and university social networks (e-mail, Facebook, Instagram, and WhatsApp) and completed an online structured questionnaire using the Google Forms web survey platform. A total of 6,525 answers were received. After excluding, aged < 18 years (*N* = 26) and respondents from other countries (*N* = 174), the final data set includedr 6,325 participants.

### Ethical Aspects

A brief description of the study and its aim as well as an informed consent were provided on the first electronic page containing the invitation to participate in the survey. All subjects consented to join in the study after clicking the “accept” icon, meaning that they have agreed with the terms of the informed consent.

This study follows the international ethical standards found in the Declaration of Helsinki (2000). All procedures involving human subjects were approved by the Research and Ethics Committee of each country as follows: Research and Ethics Committee of the Adventist University of River Plate School of Medicine in Argentina (Resolution # 1.7/2020); Ethics Committee of the Federal University of Espírito Santo in Brazil (approval #: 33948820.7.0000.5060); Ethics Committee of the University of the Americas Puebla in Mexico (approval # 019/2020); Ethics in Research Committee of the Peruvian Union University (approval # 2020-CEUPeU-00013); and Ethics Committee of the Autonomous University of Madrid, Spain (Projet: Cohorte UAM/AUF COVID-19 (CEI 106- 2082).

### Assessment of Socio-Demographic, Diet, Health and Well-Being, and Lifestyle Exposures

Data collection was carried out through a single structured digital self-administered questionnaire elaborated by the authors using the Google Forms tool. The instrument was designed and culturally adapted by a team of nutrition and lifestyle research experts to be applied in the studied countries and included questions on sociodemographic, confinement, diet, health and well-being, and lifestyle exposures. The following socio-demographic variables were evaluated: age in years; sex (male or female); marital status (single, married, separated/divorced, or widowed); educational level (elementary, middle school, high school, college, or graduate); current occupation (student, worker, housewife, unemployed, or retired); labor condition (formal worker with contract, self-employed, or public service employee). The instrument included the following questions on confinement during the pandemic: (1) *Were you part of the confinement?* The answer options were: *yes, I am still confined, yes but I have returned to work, or no;* (2) *For how many days were you in confinement/are you still in confinement?* The possible answers were: <*15, 15–30, 31–45, 45–60 days*, or *more than 61 days*; (3) *How many people lived in your home before confinement?* (4) *How many people lived in your home during confinement?*

Two questions were used to evaluate consumption of meals: (1) *Meals usually consumed before confinement: breakfast, small morning snack, lunch, small afternoon snack, dinner, and snacking between meals* and (2) *Meals usually consumed during confinement: breakfast, small morning snack, lunch, small afternoon snack, dinner, and snacking between meals*. The answer options were *yes* or *no*, for each meal. Thus, the following responses were obtained: unchanged consumption of the meal, increased consumption of the meal and decreased consumption of the meal due to COVID-19-induced confinement.

Previous and current consumption of food groups (vegetables, fruits, legumes, nuts, fish, red meat and poultry, eggs, milk, yogurt, bakery products, salty chips, soft drinks, beer, wine, distilled alcoholic beverages, and fast food) were evaluated with two questions, respectively: (1) *Choose the option that describes your consumption before confinement* and (2) *Choose the option that describes your consumption during confinement*. For each food group there were six options of frequency of consumption: never /seldom, 1–2 times a week, 3–4 times a week, 5–6 times a week, and 1 time per day, and 2+ times per day. Frequencies of intake were converted to weekly equivalents, as follows: never/seldom = zero/week, 1–2 times a week = 1.5/week, 3–4 times a week = 3.5/week, 5–6 times a week = 5.5/week. Answers of 1 time per day and 2+ times per day were collapsed for analyses and converted into weekly equivalents: 1+ times per day = 7+/week. Weekly equivalents were used to calculate the difference between consumption before confinement and during the confinement of each food group, thus generating the variable “difference in the consumption.”

For the lifestyle variables, we evaluated sleep hours/night, sleep changes (no change, sleeping more or less) and the practice of physical activity during confinement. The first question used to assess the practice of physical activity was: *During the period of confinement do you practice some type of physical activity?* The answer options were *yes* or *no*. Those who answered affirmatively were asked two more questions: (1) *How many days per week?*, with answer options ranging from 1 to 7 and (2) *What is the duration of physical activity?*, with the following answer options: <*30 min, 30 min*−*1 h, 1–2 h, or Other* (open answer).

Two questions were used to explore feelings of anxiety among participants. We asked if confinement generated feelings of anxiety (yes or no) and if feelings of anxiety were generated by other situations, such as: unemployment, illness (own or in the family), worries about pandemic information, or other cause (to be completed by the participant as an open question). Changes in lifestyle habits and eating patterns during confinement were assessed retrospectively using the same instrument which included questions about the habit before the confinement and during the confinement, separately. Self-reported height and current weight were used to calculate body mass index (BMI) in kg/m^2^. Participants were asked if there was any weight change during confinement reported either as weight gain, loss, maintenance, or ignored. Self-reported data on COVID-19 diagnostic and hospitalization were also collected.

### Data Analysis

In the present study, the percentage of individuals consuming or not consuming each meal before and during confinement due to COVID-19 in different countries was compared using the McNemar test. K-means cluster analysis was used to determine patterns of dietary changes during confinement. Variables used to create patterns were “difference in consumption” of food groups (vegetables, fruits, legumes, nuts, fish, red meat and poultry, eggs, milk, yogurt, bakery products, salty chips, soft drinks, beer, wine, distilled alcoholic beverages, and fast food). Three best interpretable patterns were created: (1) Healthier, characterized by increased consumption of fruits, vegetables, and legumes and less consumption of bakery products, and snacks, (2) No change, characterized by relatively stable (Constant) dietary patterns during the confinement compared to previous time; and (3) Less healthy, characterized by decreased consumption of vegetables, fruits, legumes, nuts, eggs, milk and yogurt during the confinement.

The chi-square test was used to analyze associations of socio-demographic, lifestyle and anxiety variables among patterns of dietary changes (healthier, no change, and less healthy) and among individuals with/without a confirmed diagnosis of COVID-19. Moreover, stepwise multivariate logistic regression analyses were performed to analyze the factors that influenced the odds of assignment to the (1) healthier, (2) no change/constant, and (3) less healthy patterns of dietary changes. The created models included socio-demographics (sex, age, educational level, employment status, and country, confinement status, confirmed diagnosis of COVID-19, and BMI category) and lifestyle factors (physical activity, sleep changes, and feelings of anxiety during confinement). The results of logistic regression analyses were expressed as odds ratio (OR) and 95% confidence intervals (95% CI). For all analyses, *p* ≤ 0.05 was considered significant. All statistical analyses were performed using the SPSS 23.0.

## Results

The studied sample consisted of 6,325 respondents with women predominating significantly (Table 1). The largest group was from Brazil and the smallest group was from Mexico. About half of the population aged 18–29 years and 73.4% of respondents had a higher educational level or more. Nearly 90% of participants were either students, workers, or both. During the period of the pandemic, the largest percentage was confined and had no confirmed diagnosis of COVID-19.

**Table 1:** Socio-demographic, confinement, diagnosis of COVID 19 and nutritional status characteristics, according to direction of changes in eating patterns during the confinement due to COVID-19 pandemic.

		**Changes in eating pattern**			
	**Total 6,325**	**Healthier** ***n* (%)** **1,435 (22.7)**	**No change** ***n* (%)** **3,894 (61.6)**	**Less healthy** ***n* (%)** **996 (15.7)**	***p***
**Sex**					
Male	2,019 (31.9)	385 (19.1)	1,329 (65.8)	305 (15.1)	<0.001
Female	4,306 (68.1)	1,050 (24.4)	2,566 (59.6)	690 (16.0)	
**Country**					
Argentina	1,111 (17.5)	320 (28.8)	637 (57.3)	154 (13.9)	<0.001
Brazil	2,171 (34.3)	573 (26.4)	1,278 (58.9)	320 (14.7)	
Mexico	686 (10.8)	154 (22.4)	384 (56.0)	148 (21.6)	
Peru	1,174 (18.5)	193 (16.4)	753 (64.1)	228 (19.4)	
Spain	1,183 (18.7)	195 (16.5)	843 (71.3)	145 (12.3)	
**Age (years)**					
18–29	3,059 (48.4)	751 (24.6)	1,761 (57.6)	547 (17.9)	<0.001
30–49	2,310 (36.5)	529 (22.9)	1,429 (61.9)	352 (15.2)	
≥50	956 (15.1)	155 (16.2)	705 (73.7)	96 (10.0)	
**Marital status**					
Single	3,599 (56.9)	865 (24.0)	2,113 (58.7)	621 (17.3)	<0.001
Married	2,360 (37.3)	495 (21.0)	1,541 (65.3)	324 (13.7)	
Separated/divorced/widowed	366 (5.7)	75 (20.5)	241 (65.8)	50 (13.7)	
**Educational level**					
High school or less	1,683 (26.6)	377 (22.9)	1,048 (62.3)	257 (15.6)	0.064
College	2,712 (42.9)	579 (21.3)	1,704 (62.8)	429 (15.8)	
Graduate	1,930 (30.5)	479 (24.8)	1,143 (59.2)	308 (16.0)	
**Employment status**					
Unemployed/retired	502 (7.9)	94 (18.7)	337 (67.1)	71 (14.1)	<0.001
Housewife	170 (2.7)	39 (22.9)	108 (63.5)	23 (13.5)	
Student	2,230 (35.3)	552 (24.8)	1,288 (57.8)	390 (17.5)	
Worker and student	1,000 (15.8)	248 (24.8)	594 (59.4)	158 (15.8)	
Worker	2,391 (37.8)	496 (20.7)	1,547 (64.7)	348 (14.6)	
Confinement^*^					
No	452 (8.8)	95 (21.0)	309 (68.4)	48 (10.6)	<0.001
Yes, I am still	1,152 (22.4)	269 (23.4)	691 (60.0)	48 (10.6)	
Yes, but I am back to my activities	3,541(68.8)	877 (24.8)	2,054 (58.0)	610 (17.2)	
Confirmed diagnosis of COVID-19^*^					
No	5,891(93.5)	1,355 (23.0)	3,619 (61.4)	917 (15.6)	0.039
Yes	405 (6.4)	73 (18.0)	257 (63.5)	75 (18.5)	
Perception of weight change during lockdown^*^					
Yes, weight gain	2,794 (48.6)	809 (29.0)	1,512 (54.1)	473 (16.9)	<0.001
Yes, weight loss	1,331 (23.1)	198 (14.9)	912 (68.5)	221 (16.6)	
No, weight maintenance	1,619 (28.1)	306 (18.9)	1,117 (69.0)	196 (12.1)	
Nutritional status^*^					
Underweight	2,017 (32.3)	451 (22.4)	1,253 (62.1)	313 (15.5)	0.941
Normal	3,380 (54.1)	775 (22.9)	2,062 (61.0)	543 (16.1)	
Overweight/obesity	844 (13.6)	196 (23.2)	515 (61.0)	133 (15.8)	

*n, number; Chi-square test*,

* *different sample size*.

Most participants reported no change in their eating habits during the pandemic (61.6%), however, most of those who changed, did so in the direction of healthier dietary choices (22.7%). The same pattern was mainly found among women, Argentinians, Brazilians, among youngest participants (<30 years), students, workers and students, among those who did confinement, and those who gained weight. No statistical significance was found between dietary changes and educational levels or nutritional status (Table 1).

When analyzing eating behavior changes in individuals from different countries, it was evident that higher proportions of Argentinians (28.8%) and Brazilians (26.4%) adopted healthier patterns of food choices during the pandemic. Spaniards followed the same trend with 16.5% individuals shifting diet patterns toward healthier options and 2.3% individuals moving in the opposite direction. Similar proportions of Mexicans adopted healthier (22.4%) and less healthy (21.6%) eating patterns, while higher proportions of Peruvians adopted less healthy eating patterns (19.4%) than those who made healthier choices (16.4%) (Table 1).

**Figure 1:**
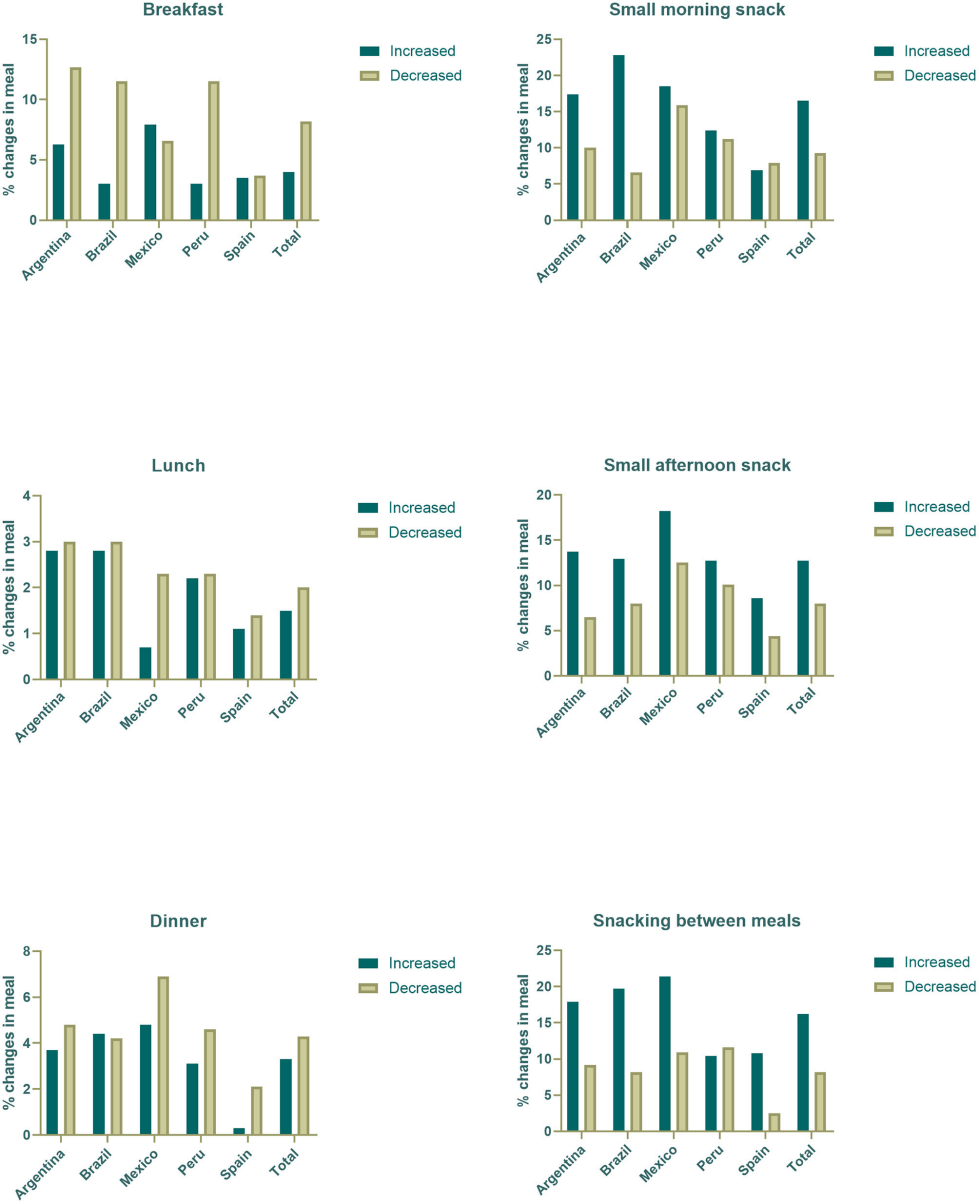
Percentages of change in meal in Iberoamerican countries.

Most participants remained constant in their pattern of meal consumption, neither introducing nor omitting meals during confinement. Respondents who reported changes are described in Figure 1. There was a trend in reducing the consumption of main meals (breakfast, lunch, and dinner) during the confinement. In contrast, participants were more likely to increase their consumption of small morning and afternoon meals. Except for Peru, they also tended to snack more between meals. The *p-*values for comparisons using the McNemar test were significant for all meals (*p* < 0.001 for breakfast, dinner, morning and afternoon meals, and snacks and *p* < 0.005 for lunch).

Table 2 shows that most participants reported doing physical activities (57.1%) 1–6 days/week (51.3%) with a duration of 30 min−1 h (62%). Changes in sleeping habits and feelings of anxiety during the pandemic were also frequently reported (72.1 and 63.0%, respectively). Interestingly, participants who reported not having changed sleeping hours during confinement slept on average 7.3 ± 1.1 h. Those who reported sleeping more had an average of 8.5 ± 1.5 h of sleep and those who reported sleeping less had an average of 6.0 ± 1.1 h of sleep (*p* < 0.001). Healthier eating choices were mainly observed among those who reported not doing physical activity or practiced fewer days/week (1–3 days/week), those who had sleep duration changes, and those who reported feeling anxious during confinement (specially feelings of anxiety from illness of family members and due to COVID-19 statistics). The sensitivity analysis for participants with COVID is reported in Supplementary Table 1.

**Table 2:** Physical activity, sleep changes and anxiety, according to direction of changes in eating patterns during the confinement due to COVID-19 pandemic.

		**Changes in eating pattern**			
	**Total 6,325**	**Healthier** ***n* (%)** **1,435 (22.7)**	**No change *n* (%) 3,894 (61.6)**	**Less healthy** ***n* (%)** **996 (15.7)**	***p***
**Physical activity during confinement**					
No	2,714 (42.9)	740 (27.3)	1,555 (57.3)	419 (15.4)	<0.001
Yes	3,611 (57.1)	695 (19.2)	2,340 (64.8)	576 (16.0)	
Physical activity during confinement (days/week)^*^					
1–3	1,710 (27.0)	365 (21.3)	1,089 (63.6)	256 (14.9)	
3–6	1,539 (24.3)	280 (18.1)	989 (64.2)	270 (17.5)	
7	362 (5.7)	50 (13.8)	262 (72.3)	50 (13.8)	
Physical activity confinement (time/week)^*^					
<30 min	623 (22.1)	135 (21.7)	379 (60.8)	109 (17.5)	<0.001
30 min−1 h	1,746 (62.0)	358 (20.5)	1,082 (62.0)	306 (17.5)	
1–2 h	446 (15.8)	96 (21.5)	286 (64.1)	64 (14.3)	
Sleep changes during confinement^*^					
No	1,754 (27.8)	305 (17.4)	1,227 (70.0)	222 (12.7)	<0.001
Yes, sleep more	2,629 (41.7)	662 (25.2)	1,521 (57.9)	446 (17.0)	
Yes, sleep less	1,918 (30.4)	462 (24.1)	1,132 (59.0)	324 (16.9)	
**Feelings of anxiety during confinement**					
No	2,327 (37.0)	416 (17.9)	1,589 (68.3)	322 (13.8)	<0.001
Yes	3,969 (63.0)	1,012 (25.5)	2,287 (57.6)	670 (16.9)	
Feelings of anxiety about work and economics	370 (9.1)	80 (20.5)	221 (59.8)	69 (19,7)	0.363
Feelings of anxiety for own or family members' disease	1,361 (34.3)	360 (26.5)	759 (55.8)	242 (17.7)	<0.001
Feelings of anxiety due to COVID-19 statistics	2,276 (57.3)	616 (27.1)	1,296 (56.9)	364 (16.0)	<0.001
Feelings of anxiety about studies	367 (9.2)	77 (21.0)	224 (61.0)	66 (18.0)	0.486
Feelings of anxiety about living together	321 (8.1)	75 (23.4)	203 (63.2)	43 (13.4)	0.514
Other sources of anxiety feelings (pregnancy, breastfeeding, addictions)	574 (14.5)	134 (23.3)	349 (60.8)	91 (15.9)	0.733

*n, number; Chi-square test*,

* *different sample size*.

Table 3 presents selected variables among participants who changed their eating pattern toward healthier choices during the COVID-19 pandemic using three multiple logistic regression models with progressive adjustments. It was found a positive association with the adoption of healthier dietary changes among those who were younger (<30 years, OR: 1.61; 95% CI: 1.2–2.1; 30–49 years, OR: 1.41; 95% CI: 1.1–1.7) and those who changed their sleep pattern, either sleeping more (OR: 1.40; 95% CI: 1.2–1.7) or less (OR: 1.46; 95% CI: 1.2–1.7). On the contrary, individuals with lower educational levels, Peruvians and Mexicans were less likely to adopt healthier diet changes (OR: 0.70 95% CI: 0.5–0.8; OR: 0.51; 95% CI: 0.4–0.6; and 0.59; 95% CI: 0.4–0.8, respectively).

**Table 3:** Comparison of socio-demographic and selected variables among participants who changed their eating patterns toward healthier choices during the COVID-19 pandemic (*n* = 1,435).^a^.

	**Model 1 OR (95% CI)**	**Model 2** **OR (95% CI)**	**Model 3 OR (95% CI)**
**Sex**			
Male	1	1	1
Female	1.28 (1.1–1.4)	1.19 (1.0–1.3)	1.23 (1.0–1.4)
**Age (years)**			
18–29	1.66 (1.2–2.1)	1.61 (1.2–2.2)	1.61 (1.2–2.1)
30–49	1.50 (1.1–1.8)	1.43 (1.1–1.8)	1.41 (1.1–1.7)
≥50	1	1	1
**Marital status**			
Single	1	1	1
Married	0.94 (0.7–1.1)	0.95 (0.7–1.1)	0.95 (0.7–1.1)
Separated/divorced/widowed	0.97 (0.7–1.3)	0.97 (0.6–1.4)	0.92 (0.6–1.2)
**Educational level**			
High school or less	0.72 (0.6–0.8)	0.72 (0.6–0.8)	0.70 (0.5–0.8)
College	0.71 (0.6–0.8)	0.73 (0.6–0.8)	0.69 (0.5–0.8)
Graduate	1	1	1
**Employment status**			
Unemployed/retired	0.95 (0.7–1.2)	1.04 (0.7–1.4)	0.96 (0.7–1.3)
Housewife	0.97 (0.6–1.4)	1.01 (0.6–1.5)	0.97 (0.6–1.4)
Student	1.09 (0.8–1.3)	1.07 (0.8–1.3)	1.10 (0.9–1.3)
Worker and student	1	1	1
Worker	0.90 (0.7–1.1)	0.86 (0.7–1.0)	0.89 (0.7–1.0)
**Country**			
Brazil	1.13 (0.8–1.3)	1.13 (0.8–1.4)	1.14 (0.9–1.3)
Peru	0.59 (0.4–0.7)	0.59 (0.4–0.7)	0.51 (0.4–0.6)
Mexico	0.64 (0.5–0.8)	0.63 (0.4–0.8)	0.59 (0.4–0.8)
Argentina	1	1	1
Spain	0.60 (0.1–1.5)	1.31 (0.1–1.5)	1.55 (0.1–1.6)
**Confinement**			
No		0.82 (0.6–1.0)	0.85 (0.6–1.1)
Yes, still confined		1	1
Yes, back to activities		0.99 (0.8–1.1)	1.02 (0.8–1.1)
**Confirmed diagnosis of COVID- 19**			
No		1	1
Yes		0.93 (0.6–1.2)	0.89 (0.6–1.2)
**Nutritional status**			
Underweight			0.91 (0.7–1.0)
Normal			1
Overweight/obesity			0.97 (0.7–1.2)
**Physical activity during confinement**			
No			1
Yes			0.68 (0.5–1.1)
**Sleep changes during confinement**			
No			1
Yes, I sleep more			1.40 (1.2–1.7)
Yes, I sleep less			1.46 (1.2–1.7)
**Feelings of anxiety during confinement**			
Not			1
Yes			1.7 (1.1–1.4)

*95% CI, 95% confidence interval; OR, Odds Ratio*.

a *Estimated by stepwise multiple logistic regression. Model 1 adjusted for socio-demographic variables (sex, age, marital status, educational level, and country); Model 2 adjusted for socio-demographic variables, confinement, and diagnosis of COVID-19; Model 3 adjusted for sociodemographic variables, confinement, diagnosis of COVID-19, nutritional status, and lifestyle (physical activity, sleep changes and anxiety during confinement) variables*.

Table 4 shows a comparison of the studied variables among participants who during the COVID-19 pandemic remained constant on their eating pattern. After adjustments, positive associations were found for educational level, lockdown and physical activity, and negative associations were observed for gender, age, diagnosis of COVID-19, sleep pattern and feelings of anxiety. Indeed, individuals with low educational levels were more likely to maintain their eating patterns than participants with graduate educational level (high school or less, OR: 1.33; 95% CI: 1.1–1.5; college, OR: 1.36; 95% CI 1.1–1.6). The absence of lockdown was also associated with no changes in eating patterns (1.43; 95% CI 1.1–1.8). The same pattern was observed among those who were physically active during the pandemic confinement (1.29; 95% CI 1.1–1.4). In contrast, women (OR = 0.88; 95% CI: 0.6–0.9), individuals with <30 years (OR: 0.55; 95% CI: 0.4–0.7), and 30–49 years (OR: 0.72; 95% CI: 0.5–0.9), with diagnosis of COVID-19 (OR: 0.77; 95% CI: 0.5–0.9), with change in sleep pattern (increased sleep time, OR: 0.68; 95% CI: 0.5–0.8 and decreased sleep time, OR: 0.67; 95% CI: 0.5–0.8), and who reported feelings of anxiety (OR: 0.72; 95% CI: 0.6–0.8) were less likely to keep eating patterns constant.

**Table 4:** Comparison of selected variables among participants who remained constant on their eating pattern during the COVID-19 pandemic (*n* = 3,849).^a^.

	**Model 1 OR (95% CI)**	**Model 2 OR (95% CI)**	**Model 3 OR (95% CI)**
**Sex**			
Male	1	1	1
Female	0.77 (0.6–0.8)	0.77 (0.6–0.8)	0.88 (0.6–0.9)
**Age (years)**			
18–29	0.50 (0.4–0.6)	0.53 (0.4–0.6)	0.55 (0.4–0.7)
30–49	0.62 (0.5–0.7)	0.69 (0.5–0.8)	0.72 (0.5–0.9)
≥50	1	1	1
**Marital status**			
Single	1	1	1
Married	1.07 (0.9–1.2)	1.2 (1.0–1.5)	1.01 (0.8–1.1)
Separated/divorced/widowed	0.95 (0.7–1.2)	1.3 (1.1–1.5)	1.05 (0.7–1.4)
**Educational level**			
High school or less	1.33 (1.1–1.5)	1.29 (1.0–1.5)	1.33 (1.1–1.5)
College	1.38 (1.1–1.6)	1.32 (1.1–1.5)	1.36 (1.1–1.6)
Graduate	1	1	1
**Employment status**			
Unemployed/retired	0.97 (0.7–1.2)	0.93 (0.6–1.2)	0.92 (0.6–1.2)
Housewife	1.10 (0.7–1.2)	1.12 (0.7–1.6)	1.11 (0.7–1.6)
Student	0.93 (0.7–1.1)	0.98 (0.8–1.1)	0.96 (0.8–1.1)
Worker and student	1	1	1
Worker	1.05 (0.8–1.2)	1.11 (0.9–1.3)	1.09 (0.9–1.3)
**Country**			
Brazil	0.95 (0.8–1.1)	0.95 (0.8–1.1)	0.91 (0.7–1.1)
Peru	1.24 (1.0–1.4)	1.22 (1.0–1.4)	1.19 (1.0–1.4)
Mexico	1.03 (0.8–1.2)	1.03 (0.8–1.2)	1.01 (0.8–1.2)
Argentina	1	1	1
Spain	1.58 (1.3–1.8)	0.87 (0.5–14.0)	0.71 (0.4–11.0)
**Confinement**			
No			1.43 (1.1–1.8)
Yes, still confined			1
Yes, back to activities			0.98 (0.8–1.1)
**Confirmed diagnosis of COVID-19**			
No		1	1
Yes		0.72 (0.5–0.9)	0.77 (0.5–0.9)
**Nutritional status**			
Underweight			1.10 (0.9–1.2)
Normal			1
Overweight/obesity			0.94 (0.7–1.1)
**Physical activity during confinement**			
No			1
Yes			1.29 (1.1–1.4)
**Sleep changes during confinement**			
No			1
Yes, sleep more			0.68 (0.5–0.8)
Yes, sleep less			0.67 (0.5–0.7)
**Feelings of anxiety during confinement**			
No			1
Yes			0.72 (0.6–0.8)

*95% CI, 95% Confidence interval; OR, Odds Ratio*.

a *Estimated by stepwise multiple logistic regression. Model 1 adjusted for sociodemographic variables (sex, country, age, marital status, educational level, and employment status); Model 2 adjusted for sociodemographic variables, confinement and confirmed diagnosis of COVID-19; Model 3 adjusted for sociodemographic, confinement, COVID-19 diagnosis, lifestyles variables (nutritional status, physical activity, and sleep changes during confinement), and anxiety during confinement*.

A comparison of chosen variables among participants who changed their eating patterns toward a less healthy profile during the COVID-19 pandemic is presented in Table 5. Positive associations were only found for age group, country of residence, diagnosis of COVID-19, and feelings of anxiety. Individuals below 30 years old (OR: 1.60, 95% CI:1.1–2.3), Peruvians (OR: 1.38; 95% CI: 1.1–1.7), Mexicans (OR: 1.61; 95% CI: 1.2–2.1), individuals with a diagnosis of COVID-19 (OR: 1.72; 95% CI 1.2–2.3), and those who reported feelings of anxiety during confinement (OR = 1.21; 95% CI: 1.1–1.4) were more likely to make dietary changes to a less healthy pattern.

**Table 5:** Comparison of selected variables among participants who changed their eating patterns toward less healthy choices during the COVID-19 pandemic (*n* = 996).^a^.

	**Model 1 OR (95% CI)**	**Model 2** **OR (95% CI)**	**Model 3 OR (95% CI)**
**Sex**			
Male	1	1	1
Female	1.14 (0.9–1.3)	1.24 (1.0–1.4)	1.22 (1.0–1.4)
**Age (years)**			
18–29	1.8 (1.3–2.4)	1.61 (1.1–2.3)	1.60 (1.1–2.3)
30–49	1.4 (1.1–1.9)	1.25 (0.9–1.7)	1.24 (0.9–1.7)
≥50	1	1	1
**Marital status**			
Single	1	1	1
Married	0.95 (0.7–1.1)	1.01 (0.8–1.2)	1.01 (0.8–1.2)
Separated/divorced/widowed	1.13 (0.7–1.6)	1.00 (0.6–1.5)	0.98 (0.6–1.5)
**Educational level**			
High school or less	0.90 (0.7–1.1)	0.97 (0.7–1.2)	0.97 (0.7–1.2)
College	0.86 (0.7–1.0)	0.92 (0.7–1.1)	0.91 (0.7–1.1)
Graduate	1	1	1
**Employment status**			
Unemployed/retired	1.09 (0.7–1.5)	1.07 (0.7–1.5)	1.08 (0.7–1.5)
Housewife	0.86 (0.5–1.4)	0.80 (0.4–1.3)	0.83 (0.4–1.4)
Student	0.99 (0.7–1.2)	0.92 (0.7–1.1)	0.93 (0.7–1.2)
Worker and student	1	1	1
Worker	1.03 (0.8–1.3)	0.99 (0.7–1.2)	1.01 (0.7–1.2)
**Country**			
Brazil	0.89 (0.7–1.1)	0.93 (0.7–1.1)	0.93 (0.7–1.1)
Peru	1.30 (1.1–1.5)	1.35 (1.1–1.6)	1.38 (1.1–1.7)
Mexico	1.57 (1.2–2.0)	1.60 (1.2–2.1)	1.61 (1.2–2.1)
Argentina	1	1	1
Spain	0.84 (0.6–1.0)	0.90 (0.6–1.1)	0.90 (0.6–1.1)
**Lockdown**			
No		0.57 (0.4–0.8)	0.59 (0.4–0.8)
Yes, still confined		1	1
Yes, back to activities		0.97 (0.8–1.1)	0.98 (0.8–1.1)
**Confirmed diagnosis of COVID-19**			
No		1	1
Yes		1.75 (1.2–2.4)	1.72 (1.2–2.3)
**Nutritional status**			
Low weight			0.94 (0.7–1.1)
Normal			1
Overweight/obesity			1.14 (0.8–1.4)
**Physical activity during confinement**			
No			1
Yes			1.04 (0.8–1.2)
**Sleep changes during confinement**			
No			1
Yes, sleep more			1.18 (0.9–1.4)
Yes, sleep less			1.23 (0.9–1.5)
**Feelings of anxiety during confinement**			
No			1
Yes			1.21 (1.1–1.4)

*95% CI, 95% Confidence interval; OR, Odds Ratio*.

* *Estimated by stepwise multiple logistic regression. Model 1 adjusted for sociodemographic variables (sex, country, age, marital status, educational level, and employment status); Model 2 adjusted for sociodemographic variables, confinement and confirmed diagnosis of COVID-19; Model 3 adjusted for sociodemographic, confinement, COVID-19 diagnosis, lifestyles variables (nutritional status, physical activity, and sleep changes during confinement), and anxiety during confinement*.

## Discussion

During confinement due to the first wave of COVID-19 pandemic, most participants from the five Ibero-American studied countries remained constant. This trend was greater in Spain. Among those who changed, the change toward a healthier dietary pattern prevailed, especially in Argentina and Brazil, with an increase in the frequency of consumption of fruits, vegetables, legumes and a decrease in snacks and bakery products. Peruvians and Mexicans were less likely to make healthy changes in food consumption when compared to Argentinians. The pandemic situation also resulted in a constant pattern of meal consumption, neither introducing nor omitting meals during confinement. Nevertheless, among those who changed meal patterns, there was a trend in reducing the consumption of main meals (breakfast, lunch, and dinner) and in increasing the consumption of small meals and snacks in all studied countries More than half of the participants affirmed to be doing physical activity at home. Changes in sleep pattern, feelings of anxiety and perception of weight gain were also reported. Individuals who showed sleep changes were less likely to remain constant and more likely to modify their diets to a healthier pattern. In contrast, individuals with a confirmed diagnosis of COVID-19, and those who reported feeling anxious during confinement were more likely to perform changes to a less healthy eating pattern.

Other studies have also found a no change pattern in eating habits during the first wave of the COVID-19 pandemic, among older persons from the general Finnish population (20) and Polish adults (10). Scientific reports carried out before the pandemic revealed that diet quality in the studied Ibero-American countries deserves attention and surveillance. Therefore, our results showing maintenance of dietary patterns in most participants during the pandemic can be worrying. The Latin American region is facing an ongoing epidemiological nutritional transition. Data from the ELANS study (Latin American Study of Nutrition and Health) carried out just before the pandemic with an urban sample of 9,218 individuals between 15 and 65 years old from eight Latin American countries (Argentina, Brazil, Chile, Colombia, Costa Rica, Ecuador, Peru, and Venezuela) has shown that diet quality is deficient and with low diversity (21, 22). Only 7.2% of the overall sample reached the WHO's recommendation for fruits and vegetable consumption (400 g/day). Less than 3.5% of the sample met the optimal consumption levels of vegetables, nuts, whole grains, fish and yogurt.

In Mexico poor diets were the third leading risk factor for disability-adjusted life Years (DALYs) among adults (10.6%), followed by high BMI (11.7%) and high blood fasting glucose (11.1%), both of which are also partially linked with dietary habits (23). The intakes of sugar-sweetened beverages and energy-dense nutrient-poor foods are also especially problematic in Mexico (24).

Likewise, a Spanish national survey on the trends and evolution of patterns of food consumption showed changes in energy and nutrient intake from 1964, 1981, 1991, to 2000–2012, differing from the traditional and healthy *Mediterranean Diet*. Meat and derived product consumption was higher than the recommendations, whereas for cereals and their derivatives, vegetables and greens, fruits, and legumes and pulses, consumption was below recommendations for the Spanish population. Some staple Mediterranean foods (e.g., bread and olive oil) showed a dramatic decline (25).

Therefore, changes toward a higher quality of dietary habits would be important for Ibero-American countries, especially considering the context of the current pandemic. It is well-known that the regular intake of foods with high nutritional values is associated with fewer ailments and better health conditions. Studies show that consistent eating of whole food plant-based diets may assist the immune system in fighting human pathogens by improving the intestinal microbiota and providing minerals, vitamins, and several other phytochemicals essential for the proper functioning of human metabolism (26, 27). Fruits and vegetables are rich in fibers supporting a healthy gut microbiome, and antioxidant phytochemicals such as alkaloids and flavonoids. Investigations have shown that certain types of vegetables may exert anti-inflammatory, antioxidant, cytoprotective, and antiviral properties due to elements found in garlic, onions, ginger, Curcuma, and berries (28). The promotion of changes in this direction could potentially prevent the future onset of chronic diseases and strengthen the organism to fight against infections.

Among participants who changed their food choices most went toward a healthier pattern. This result was also reported in studies performed in Poland (10), Italy (18), and Finland (20), during the COVID-19 pandemic. Indeed, spending more time at home may increase cooking time and promote better adaptation to healthier nutritional standards (29). Busy routines that made it difficult to consume homemade meals and led to the consumption of high-calorie fast foods, might have been replaced by homemade healthier preparations for those participants, during confinement. This behavior may also be related to COVID-19 driven stay-at-home guidelines. To support healthy food intakes during self-quarantine and isolation, the WHO published a document that recommends prioritizing legumes, fresh fruits, and vegetables and for cooking their recipes at home (30). The COVID-19 pandemic fostered the search for new foods and nutritional supplements which are safe and suitable to mitigate the infection and the subsequent state of hyper-inflammation, oxidation, and cytokines storms caused by the SARS-CoV-2 on humans, which is associated in some cases with higher health complications, multiorgan injury and worse prognosis (31).

Mexicans and Peruvians showed an inverse association for adherence to a healthier eating pattern in the present study. Data from the ELANS study showed that although Peruvians had the highest intake of fruits and whole grains and lower consumption of red and processed meat when compared with other Latin American countries, they had the highest intake of homemade sugar-sweetened beverages. Indeed, almost the entire sample of participants from Peru (97%) consumed such beverages. Moreover, Peruvians showed the highest prevalence of sedentary behavior (18, 32). Mexicans also face nutritional transition. In a study to evaluate diet changes in the Mexican population during confinement due to COVID-19, 37.2% of the participants reported changes to less healthy diet habits, with an increase in the consumption of sweets and desserts in 39% of men and 51.6% of women (33).

In our study, we observed that younger people were more likely to be classified in the group reporting changes consistent with a healthier diet. This was also observed among young Italians (18) who increased their compliance to the Mediterranean diet with higher consumption of olive oil, vegetables and legumes during confinement. This behavior shows the ability to adapt to drastic changes typical of this age. We also identified an inverse association between a healthier pattern and educational level, as seen in the USA. In this research, low-income and educated adults suffered direct effects caused by the pandemic, represented by the presence of food insecurity at all levels (34).

All countries showed a reduction in the main daily meals, particularly breakfast. This was more common in Argentina, Peru, and Brazil. There was also an increase in the consumption of intermediate meals in all countries. There is evidence that skipping breakfast increases the likelihood of obesity, which is related to the quality of life and health outcomes (35). The omission of main meals can lead to a higher energy intake in intermediate meals, and snacks throughout the day. These poor dietary habits associated with a shorter time spent in physical activity can impact the development of obesity and other chronic diseases (36).

Increased consumption of snacks was observed in all countries of our study, except for Peru. This behavior can be associated with ready-to-eat, high-calorie quick foods. Consumption of these foods is commonly associated with environmental, cognitive, and affective variables (e.g., boredom and stressful situations) which encourage their consumption outside the main meals (32, 37), such as reported during confinement by COVID-19.

Adopting and maintaining healthy lifestyle habits is recommended as a fundamental physical and mental health principle, especially during COVID-19 confinement. In our study, more than half of the participants confirmed the practice of physical activity during confinement, especially those younger than 30 years and older than 50 years of age (60% of both groups declared to do some type of physical activity at home). Similar results were observed in an Italian survey of 3,533 people with 10% of the participants reporting increased exercise training during the confinement (18). Likewise, in a Mexican population (*n* = 1,084), 53.2% of the participants practiced physical activity during confinement (33).

Our study also showed that half of the participants reported weight gain. Although we have not measured energy intake or energy expenditure, we theorize that a possible energy imbalance between food consumption and energy expenditure could be contributing to weight gain, regardless of the quality of the diet. Lower energy expenditure during the pandemic can result from a reduction of outdoor physical activities due to the implementation of mobility restrictions. Moreover, greater daily energy intake could have occurred over the pandemic confinement as reported in some (38, 39). It was estimated that during the COVID-19 lockdown there was an increase of 6% in daily energy intake in Spain (39). Other potential contributors to weight gain perceived by the participants of this study could be the irregularity of meals and the mealtime, with increased consumption of food outside of main meals (snacks and intermediate morning and afternoon meals); and higher levels of anxiety (40). It is known that anxiety is associated with poorer diet quality (41), especially during the pandemic (17). Some studies have found that snacking was used as a mechanism to help cope with increased anxiety levels during self-quarantine (38, 40, 42).

Sleep is another lifestyle habit critical to overall health. Consistent sleep strengthens the immune system (43), while sleep deprivation is connected with the risk of chronic diseases (44, 45). We found some changes in the sleep pattern in Ibero-American countries associated with sleep duration. There was an increase in the number of sleeping hours. During confinement, changes in routines and fixed sleeping times may have influenced and altered physiological regulators of sleep. Besides, most individuals were either students, workers, or both and approximately half of the participants were under 30 years of age. During confinement, many workers and students changed their activities to a remote form. This could have favored flexible hours of work or study at home, ensuing in late sleeping which may have also been associated with the reduction of breakfast intake, an unhealthy habit associated with higher mortality from cardiovascular causes (46).

In our study, changing the eating pattern toward a less healthy profile was positively associated with a confirmed diagnosis of COVID-19 and feelings of anxiety. Recent investigations showed high scores of unhealthy eating behaviors and a high-frequency of depressive and anxiety symptoms in two Mediterranean countries during the COVID-19 outbreak (17, 47). A high prevalence of anxiety generated by confinement during the pandemic was also observed in Saudi Arabia (27.7%) (48) and China (28.8%) (49). Anxiety has been directly associated with poor eating habits and inversely associated with healthy eating habits. In Greece, greater anxiety was associated with an increase in the consumption of sweets and meat products in women (50). An Iranian study found an inverse association between anxiety and higher consumption of vegetables, fruits, and dairy products (51).

This is a cross-sectional study conducted in a convenience sample during a restricted time frame, which contributes to specific limitations. The lack of representativeness and the different sample number for each country are the main limitations of our study. Response rates were different among countries due to the intrinsic characteristics of each country, and strategies of recruitment. Therefore, it is not possible to expand the results to the population of each country. Although mean age and educational level are proportionally distributed among countries, there is an over-representation of younger individuals (<30 years) and of women, reflecting the known lower interest in health-related matters among men (52). Moreover, all data collected are self-reported and this could make them not completely reliable, especially when it comes to reporting behaviors for which there may be a social stigma (e.g., alcohol and tobacco consumption). Other limitations could be a sampling bias with university students and workers being the majority of the population and the lack of information about religious practices which could have influenced diet and lifestyle changes.

Due to the temporary, unique and relatively unpredictable character of the phenomenon under study (the SARS-CoV-2 pandemic), the instrument was designed to capture information in that context and was culturally adapted by a team of nutrition and lifestyle research experts to be applied in each of the countries under study. Diet information was not used to quantify food consumption, nutrients or other components. Instead, questions concerning eating or lifestyle habits were applied to collect both information on the period before the pandemic and during the period of confinement. Such information is subject to the individual's memory, which could have caused measurement errors inherent to this type of method.

This study has some strengths. To our knowledge, this is the first study that evaluated and compared dietary and lifestyle changes among adults from Ibero-American countries (Argentina, Brazil, Mexico, Peru, and Spain) including design and implementation during the social confinement phase of the pandemic in all countries. Nevertheless, we acknowledge some distinct factors among countries (e.g., data collection during spring and early summer in Spain, late summer in Mexico and late winter in South American countries), which may have affected our results. Government policies also differed between countries concerning confinement. While in Spain and Mexico stricter restrictions were relaxed during summertime, Argentina and Peru faced stronger restrictive health protocols. Despite the growth of the pandemic curve in Brazil at that time, there was no homogeneity in governmental policies. Social distancing policies were adopted in several states, including those of which were most of the participants in this study (Espírito Santo and Rio de Janeiro). Nevertheless, regardless of the season of the year or how restrictive governmental measures could be at any time point, many people had to remain confined, and participants of this study were asked whether they were confined.

Despite its limitations, this study was performed with robust data analysis and covers a very important public health question in countries that are already at high risk of chronic diseases which are strongly affected by lifestyle and eating choices. The pandemic situation can further aggravate health status by affecting lifestyle choices. Therefore, our study provides additional information that can be used to assist guide policies to prevent deleterious consequences that may affect the incidence of chronic diseases.

## Data Availability Statement

The raw data supporting the conclusions of this article will be made available by the authors, without undue reservation.

## Ethics Statement

The studies involving human participants were reviewed and approved by Research and Ethics Committee of the Adventist University of River Plate School of Medicine in Argentina (Resolution # 1.7/2020); Ethics Committee of the Federal University of Espírito Santo in Brazil (approval #: 33948820.7.0000.5060); Ethics Committee of the University of the Americas Puebla in Mexico (approval # 019/2020); Ethics in Research Committee of the Peruvian Union University (approval # 2020-CEUPeU-00013); and Ethics Committee of the Autonomous University of Madrid, Spain (Project: Cohorte UAM/AUF COVID-19 (CEI 106- 2082). The patients/participants provided their written informed consent to participate in this study. Written informed consent was obtained from the individual(s) for the publication of any potentially identifiable images or data included in this article.

## Author Contributions

OE-M conceived the idea of this project, directed the statistical analyses, and wrote the initial draft of the paper. MCTM was an active collaborator with OE-M at each step and reviewed and edited the manuscript. MCTM, OE-M, DS, and MCBM were responsible for data collection in Brazil. TP and KL were responsible for data collection in Mexico. They conducted the statistical analyses and wrote the Methodology section with OE-M and DS. SP and FP were responsible for data collection in Argentina and reviewed the manuscript. SH-V and MR-V were responsible for data collection in Peru and SH-V reviewed the manuscript. MPM and AM-U designed the instrument and were responsible for data collection in Spain. MPM also conceived of the idea and design of this project with MCBM, reviewed the manuscript and provided funding for the publication. MCBM coordinated all steps of this project and worked closely with OE-M reviewing and editing the manuscript and the analysis. All authors participated in the project design and data collection in the different studied countries.

## Conflict of Interest

The authors declare that the research was conducted in the absence of any commercial or financial relationships that could be construed as a potential conflict of interest.
