# Supplementary material for: Diet and Lifestyle Changes During the COVID-19 Pandemic in Ibero-American Countries: Argentina, Brazil, Mexico, Peru, and Spain
Source: Front Nutr. 2021 Jun 2;8:671004. doi: 10.3389/fnut.2021.671004 (PMC8206276; doi:10.3389/fnut.2021.671004)
Supplement: Supplementary file 1 [file Data_Sheet_1.PDF]

Supplementary Table 1: Socio-demographic, confinement, nutritional status, lifestyle, anxiety, and changes in eating pattern characteristics, according to diagnosis of COVID-19 during the confinement.

	With COVID-19<br>n (%)<br>405 (6.4)	Without COVID-19<br>n (%)<br>5891 (93.6)	*p*
**Sex**			
Male	139 (34.3)	1872 (31.8)	0,288
Female	266 (65.7)	4019 (68.2)	
**Country**			
Argentina	13(3.2)	1098 (18.6)	**<0,001**
Brazil	103 (25.4)	2044 (34,7)	
Mexico	27(6.7)	659 (11.2)	
Peru	106(26.2)	1068 (18.6)	
Spain	156 (38.5)	1022 (17,3)	
**Age (years)**			
18-29	163 (40.2)	2885 (49.0)	**0,002**
30 – 49	166 (41.0)	2134 (36.2)	
≥ 50	76 (18.8)	872 (14.8)	
**Marital status**			
Single	206 (50.9)	3377 (57.3)	**0.040**
Married	173 (42.7)	2177 (37.0)	
Separated/divorced/widowed	26 (11.8)	337 (5,7)	
**Educational level**			
High school or less	81 (21.0)	1553 (28.4)	**0.004**
College	94 (24.4)	1333 (24.4)	
Graduate	211 (54.7)	2584 (47.2)	
**Employment Status**			
Unemployed/retired	31 (7.7)	468 (8.0)	**<0,001**
Housewife	9 (2.2)	161 (2.7)	
Student	95 (23.6)	2124 (36.2)	
Worker and student	66 (16.4)	928 (15.8)	
Worker	201 (50.0)	2181 (37.2)	
**Confinement***			
No	34 (13.6)	417 (8.6)	**<0,001**
Yes, I am still	87 (34.8)	1058 (21.7)	
Yes, but I am back to my activities	129 (51.6)	3396 (69.7)	
**Perception of weight change during lockdown***			
Yes, weight gain	156(38.5)	2637 (44.8)	0,364
Yes, weight loss	122 (30.1)	1207 (20.5)	
No, weight maintenance	103 (25.4)	1513 (25.7)	
**Nutritional Status****			
Underweight	120 (30.3)	1896 (32.5)	0,356
Normal	228 (57.6)	3148 (53.9)	
Overweight /Obesity	48 (12.1)	796 (13.6)	
**Physical activity during confinement**			
No	169 (41.7)	2521 (42.8)	0,678
Yes	236 (58.3)	3370 (57.2)	
**Sleep changes during confinement***			
No	108 (26.7)	1644 (27.9)	
Yes, sleep more	161 (39.8)	2466 (41.9)	0,364
Yes, sleep less	136 (33,6)	1780 (30.2)	
**Feelings of anxiety during confinement**			
No	149 (36.8)	2176 (37.0)	0,958

Yes	256 (63.2)	3709 (63.0)	
**Direction of change in eating pattern during confinement**			
Healthier	75 (18.5)	1355 (23.0)	**0,039**
No change	257 (63.5)	3619 (61,4)	
Less healthy	73 (18.0)	917 (15,6)	

n: number; Chi-square test, *different sample size
